# Acute Respiratory Barrier Disruption by Ozone Exposure in Mice

**DOI:** 10.3389/fimmu.2019.02169

**Published:** 2019-09-13

**Authors:** Milena Sokolowska, Valerie F. J. Quesniaux, Cezmi A. Akdis, Kian Fan Chung, Bernhard Ryffel, Dieudonnée Togbe

**Affiliations:** ^1^Swiss Institute of Allergy and Asthma Research, University of Zurich, Davos, Switzerland; ^2^Christine Kühne – Center for Allergy Research and Education (CK-CARE), Davos, Switzerland; ^3^Laboratory of Experimental and Molecular Immunology and Neurogenetics (INEM), UMR 7355 CNRS-University of Orleans, Orléans, France; ^4^Airways Disease, National Heart and Lung Institute, Imperial College London, London, United Kingdom; ^5^ArtImmune SAS, Artinem, Orléans, France

**Keywords:** inflammation, cell death, interleukins, mucus, tight junctions, innate immunity

## Abstract

Ozone exposure causes irritation, airway hyperreactivity (AHR), inflammation of the airways, and destruction of alveoli (emphysema), the gas exchange area of the lung in human and mice. This review focuses on the acute disruption of the respiratory epithelial barrier in mice. A single high dose ozone exposure (1 ppm for 1 h) causes first a break of the bronchiolar epithelium within 2 h with leak of serum proteins in the broncho-alveolar space, disruption of epithelial tight junctions and cell death, which is followed at 6 h by ROS activation, AHR, myeloid cell recruitment, and remodeling. High ROS levels activate a novel PGAM5 phosphatase dependent cell-death pathway, called oxeiptosis. Bronchiolar cell wall damage and inflammation upon a single ozone exposure are reversible. However, chronic ozone exposure leads to progressive and irreversible loss of alveolar epithelial cells and alveoli with reduced gas exchange space known as emphysema. It is further associated with chronic inflammation and fibrosis of the lung, resembling other environmental pollutants and cigarette smoke in pathogenesis of asthma, and chronic obstructive pulmonary disease (COPD). Here, we review recent data on the mechanisms of ozone induced injury on the different cell types and pathways with a focus on the role of the IL-1 family cytokines and the related IL-33. The relation of chronic ozone exposure induced lung disease with asthma and COPD and the fact that ozone exacerbates asthma and COPD is emphasized.

## Introduction

Human ozone (O3) exposure represents a major health issue (1, 2) playing an important role in the pathogenesis of chronic respiratory diseases such as asthma and chronic obstructive pulmonary disease (COPD). Ozone causes acute epithelial airway wall injury, inflammation, and airway hyperreactivity (AHR). Ozone elicits irritation of the airways with cough, bronchoconstriction, and inflammatory cell infiltration with loss of respiratory function. AHR represents a complex response of the airways to the release of bronchoconstrictive mediators and cholinergic stimulation, and is a hallmark of ozone exposure which is shared with allergic asthma. Furthermore, increased ozone exposure, especially occurring during thunderstorms, provokes severe exacerbations of asthma and may even contribute to the asthma-related deaths (3–7). A recent epidemiologic study revealed that even a short-term exposure to ambient air pollution such as PM2.5, O_3_, and NO_2_ significantly increased the risk of asthma mortality (8). Chronic ozone exposure leads to a progressive loss of the gas exchanging alveoli, a phenomenon known as emphysema, usually associated with chronic inflammation, fibrosis, and terminal respiratory failure, observed in patients with chronic obstructive pulmonary disease (COPD) and severe asthma (9). Of note, the pathogenesis of chronic lung diseases is complex and comprises the effects of various environmental particulates, toxins, chemical sand pollutants, detergents, respiratory viruses, microbial dysbiosis as well as allergen exposure, and is influenced by diverse genetic and epigenetic factors (10–15).

The respiratory airway epithelium forms a physical barrier and first line of defense of mucosal immunity (16, 17). Tight junctions (TJ) and adherens junctions (AJ), fluid, mucus, surfactant proteins, and motility of cilia are critical for the barrier control and innate response (18). Ozone impairs the function of critical proteins of the epithelial barrier (19), which will be discussed later. In addition, there is increased proliferation of the airway epithelial cells following exposure to ozone, likely as a result of direct oxidative epithelial damage (20).

Inflammatory cytokines such as members of the IL-1 family, including IL-1α, IL-1β, IL-18, IL-33, and IL-36 (21–23) as well others and several chemokines are upregulated upon ozone exposure and play major roles in the inflammatory and pathogenic response. IL-1 is involved in the inflammatory response (24), while IL-33 may have protective effects in ozone-induced inflammation as discussed below. We review here the most recent findings on ozone involvement in bronchiolar epithelial barrier dysfunction, acute lung injury, inflammation, resolution, and defective repair (20).

## Respiratory Barrier Integrity

The integrity of the epithelial barrier depends on tight junctions (TJ) and adherens junctions (AJ), which insure apicobasal cell polarity, but also mucus, fluid, and function of the cilia (18, 25–27). Tight junctions comprise the claudin family, occluding, and tricellulin. In addition, several scaffolding proteins, such as zonulae occludens (ZO)-1, ZO-2, ZO-3, multi-PDZ domain protein 1, and others have been identified in the tight junctions (28, 29). E-cadherin, as well as TJs were reduced in patients with asthma (30–32). Common respiratory viruses, such as human rhinovirus (HRV) (33, 34) or respiratory syncytial virus (RSV) (35) disrupt and impair airway epithelial barrier and delay healing of infected epithelium (36), through NADPH oxidase-1 and ROS-dependent mechanisms (33, 37, 38). Disruption of tight junctions with leak of the epithelium allows systemic access of irritants, pathogen, and allergens (15, 39), as well as the drainage of host proteins, lipid mediators, or cells into the airway lumen, where they may perpetuate inflammatory response, acting back on epithelium. Depending on the dose of allergen and airway inflammation, the Zo-1 and Cld-18 proteins expression are decreased in eosinophilic asthma, but it is even more pronounced in mixed and neutrophilic asthma phenotype (27). Epithelial barrier is impaired not only in the lower airways of patients with asthma (32), but also in the nasal mucosa of allergic rhinitis due to house dust mite (HDM) (40) displaying reduced occludin and ZO-1 levels. Ozone exposure disrupts tight junction proteins and hence the function of the respiratory barrier as reported recently (27, 41, 42).

## Direct Disruption of Epithelial Barrier by Ozone

**Figure 1:**
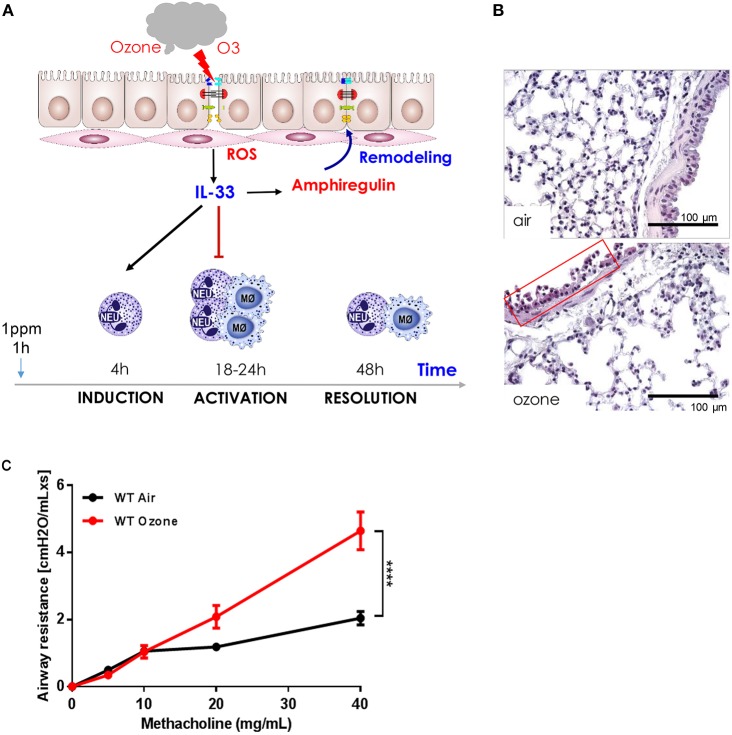
Ozone-induced epithelial barrier dysfunction, AHR, ROS production, cell recruitment, cell desquamation, remodeling, and repair in mice. **(A)** Scheme showing ozone/ROS-induced disruption of bronchiolar epithelium, cell integrity, tight junctions with protein leak, and cell death with the release of IL-33. Neutrophils and macrophages are recruited within 18 h and macrophages release protective amphiregulin (AREG) with resolution of inflammation and remodeling (43). **(B)** Micrograph of ozone-induced disruption of bronchiolar epithelium with desquamation, cell death, and focal inflammation at 18 h after exposure (1 h, 1 ppm), scale bar 100 μm. **(C)** Airways hyperreactivity (AHR): ozone enhances methacholine induced bronchoconstriction measured as resistance (RL) using invasive plethysmography 24 h after exposure (1 h, 1 ppm, unpublished data).

Ozone causes immediate damage of the bronchiolar epithelial cell barrier with cell stress, desquamation, and death with leak of protein and DNA into the airspace within 1–2 h. Since several mediators of inflammation are not yet detectable at this time, we postulate that ozone induced ROS has a direct effect on essential components of the bronchiolar cell integrity with reduced cilia function, tight junctions, mucus, and surfactant protein production (43). This first phase of ozone induced damage of the airway epithelium is followed by a second phase at of bronchiolar epithelial injury and cell death with protein leak and influx of neutrophils, ROS expressing myeloid cells, IL-1α and IL-33 production by epithelial and myeloid cells. Thus, the data suggest that ozone causes a biphasic response, an immediate direct injury via ROS and a second damage by myeloid cells, moving to and exacerbating epithelial cell damage. Indeed, neutrophil depletion by antibody against granulocytes attenuates the second phase. Similar disruptions of the respiratory barrier as shown for ozone has been described before by other air pollutants such nitrogen dioxide, disulfide, particle, chemicals, and cigarette smoke- all of which can cause chronic pulmonary diseases, but usually require longer exposure time and at higher concentrations than ozone (38, 44). A schematic view of the initial events at the respiratory barrier and early repair process is given in Figure 1.

## Disruption of Tight Junctions

Upon a single ozone exposure, we found enhanced expression of E-cadherin, ZO-1, and claudin-4 using immunofluorescence (43). By contrast, the epithelial E-cadherin, ZO-1, and Cld-4 expression are reduced in the absence of IL-33 (43). Epithelial E-cadherin expression is also reduced in IL-33/ST2 receptor deficient mice, suggesting a protective effect of IL-33. At the transcriptional level, an increased Cld-4 level (4–6 h) or E-cadherin (4–8 h) expression were found. Therefore, the data suggest that IL-33/ST2 signaling has a protective effect. This IL-33-dependant epithelial protection is probably an early defense mechanism in physiological condition. However, in the settings of allergic inflammation, excess of pathogenic Th2 cells and ILC2 in the epithelial wall, reacting to IL-33, lead to increased inflammation, and symptoms of the disease. Interestingly, in the chronic airway inflammation impairment of the barrier consists of decreased expression of several TJ proteins, but also a subsequent increase of TJs, such as CLD-4 (27). Recent reports confirm that ozone exposure impairs the function of epithelial barrier tight junction proteins (27, 41, 42).

Within 24 h after ozone exposure basal cells proliferate and at 48 h the bronchiolar epithelium is restored with firm tight junctions, mucus, and cilia (43). The mechanisms of inflammation including resolution of inflammation, remodeling of airways, and repair mechanisms are an area of intense research (13, 37, 45–47).

## Airway Hyperreactivity

Ozone exposure causes bronchoconstriction measured by the pulmonary function tests in humans and airway hyperreactivity (AHR) in rodents and humans as assessed by increased reactivity of the airways to cholinergic stimulation (44, 48). Mice exposed to ozone display a dose-dependent increased of airway resistance (RL) to methacholine aerosol as measured by invasive plethysmograph (Figure 1C). This has also been demonstrated in humans (48).

The underlying mechanisms of AHR are due to the complex response of the airway wall including effect of oxidative stress (49, 50) activation of kinase pathways, the release of chemokines, cytokines (51, 52), and lipid mediators, increased sensitivity of bronchial smooth muscle cells either directly or through an effect on innervation and neuropeptides (53), which is beyond the scope of this review.

The transient receptor potential cation channel subfamily V member1 (TRPV1) is upregulated by ozone in experimental asthma, which is sensitive to a TRPV1 antagonist. The TRPV1 antagonist suppresses the neuropeptide calcitonin gene-related peptide (CGRP) and thymic stromal lymphopoietin (TSLP) (54). Thus, TRPV1 expression may be an important mechanism for asthma exacerbation upon exposure to ozone and other environmental pollutants. Ozone induced AHR and lung inflammation is characterized by increased neutrophils recruitment in the airways and lung (54), although the role of neutrophils in inducing AHR is controversial (55, 56). Interestingly, NKT cell-deficient (CD1d^−/−^ and Jα18^−/−^) mice are resistant to ozone-induced AHR and have reduced neutrophils. Further, anti-CD1d antibody blockade of NKT cell activation prevented ozone-induced AHR. NKT cells producing IL-17 causes AHR, which was prevented in IL-17A deficient mice or by anti-IL-17 antibody blockade (57, 58). ROS mediated AHR and inflammation further depends on danger activated proteins such as HMGB1, HSPs, RAGE, and others activating the molecular pattern recognition receptors Toll-Like Receptors (TLR) 2 and 4 and the adaptor protein, MyD88 (59). However, the list of endogenous mediators activating AHR includes lipid mediators, prostaglandins and leukotrienes in response to ozone is incomplete (51, 60, 61).

These effects of ozone on bronchoconstriction and AHR raise the possibility that ozone may be involved in underlying a specific type of asthma, namely the neutrophilic inflammatory phenotype of asthma (62, 63). However, the evidence linking neutrophilic asthma to exposure to ozone as a constituent of pollution is unclear, although an increase in exacerbations of asthma in patients with asthma following a peak increase in levels of environmental ozone has been reported (64).

## Reactive Oxygen Species and Cell Death Via Oxeiptosis

**Figure 2:**
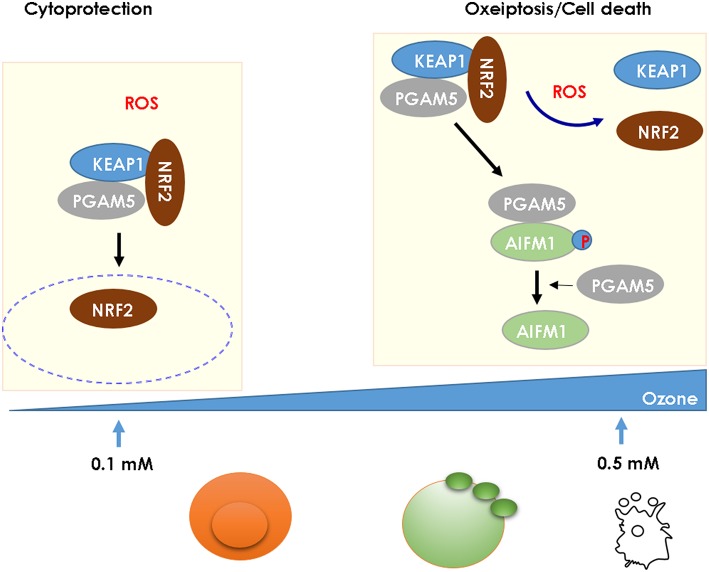
High ROS levels induce oxeiptosis, a novel cell death via the phosphatase PGAM5 activating the death effector AIFM1 in mice. While low concentration of ROS (0.1 mM) release the cytoprotective NRF2 from the KEAP1/PGAM5 complex in the cytosol, at high concentration of ROS (1 mM), the phosphatase PGAM5 is released from the mitochondrial KEAP1/NRF2/PGAM5 complex and PGAM5 dephosphorylates AIFM1, which is the effector protein defining the oxeiptosis death pathway (68).

Ozone generates reactive oxygen species (ROS), mitochondrial damage with oxidant stress activating the NLRP3 inflammasome and contributes to AHR, airflow obstruction, and emphysema (2, 65). ROS has a dual effect on cell integrity: at low concentration, it has a beneficial, cytoprotective effect, while at high concentration it is cytotoxic causing cell death. KEAP1 acts as a ROS sensor, which triggers at high ROS levels as found upon ozone exposure a novel caspase-independent cell-death pathway known as oxeiptosis (66, 67) (Figure 2). We found that ROS-induced cell-death depends on interactions of cellular ROS sensor KEAP1 and the phosphatase PGAM5 with antioxidant function and AIFM1, a pro-apoptotic factor. At high ROS concentration PGAM5 is released from the complex and activates the pro-apoptotic factor AIFM1 inducing apoptotic pathway. PGAM5 deficient mice have enhanced lung inflammation with proinflammatory cytokines upon ozone-exposure. Furthermore, in Influenza A virus infection, viral load and lung inflammation were increased in PGAM5 deficient mice, which succumbed to infection (68, 69). Oxeiptosis represents a different, non-canonical cell-death pathway, which is novel, ROS-sensitive, caspase independent, cell-death pathway important for protection against inflammation induced by ROS or viral pathogens (69). The molecular events of oxeiptosis are depicted in Figure 2. Other cell-death pathways include inflammasome-mediated apoptosis and pyroptosis, but also necroptosis and ferroptosis. Their relative roles of these pathways need to be further investigated in the ozone model.

## Inflammatory Cells Recruited in the Lung

Cell and tissue damage induce inflammatory cell recruitment including neutrophils, macrophages, innate lymphoid cells, and more. Neutrophils play a critical role in acute and chronic inflammation (70). Neutrophils migrate within and through the vessels depending polarization within activated venules activate platelets present in the bloodstream (71). Neutrophils scan for activated platelets resulting in the redistribution of receptors via the selectin ligand PSGL-1 that drive neutrophil migration allowing the interaction with the endothelium and the circulation before inflammation proceeds. Thus, a close interaction between neutrophils and platelets drives neutrophil migration to sites of injury or cell death. We have described the inflammatory response upon ozone exposure (24). Upon a single ozone exposure at 1 ppm for 1 h the analysis at 4 h after ozone exposure revealed desquamation of epithelial cells with increased CXCL1, CCL2/MIP-2, and IL-6 production, which was followed by increased macrophages and neutrophils in BALF at 6 h. At 24 h, the inflammatory response was further enhanced in the absence of ST2 or IL-33 with predominant neutrophils and interstitial macrophages in the lung. We investigated the role in the second inflammatory phase by GR-1 antibody depletion of neutrophils and other myeloid cells. GR-1 cell depletion with anti-GR1 antibody reduced protein leak, myeloid and epithelial cell in BALF and reduced parenchymal injury and inflammation with reduced MMP9 and increased amphiregulin expression as sign of tissue repair. Therefore, neutrophils contribute to tissue injury and repair as reported in other inflammatory models (72).

Besides T lymphocytes not discussed here, innate lymphocytes (ILC) are involved in the early immune response (54, 73, 74). Tissue-resident ILC-2 is involved in both physiologic and pathologic responses, yet their physical tissue niches are poorly described (75). ILC2 are recruited upon ozone exposure, but there is little information available on their roles in inflammation and airway hyperreactivity (6, 43, 76, 77).

Natural killer T cells expressing IL-17 may induce airway hyperreactivity, which depended on IL-17 expression as mentioned before (57). Platelets are likely involved in ozone induced neutrophilic inflammation, but no studies address their role in ozone induced tissues injury. The role of platelets has been recently studied in allergic inflammation (78).

Fibroblast, stromal cells, pericytes, and endothelial cells are potential targets of ozone (79, 80), which is an area for further investigations. A recent study defined a perivascular fibroblast-like stromal cells producing IL-33 and TSLP which regulate ILC2s and type 2 immunity (75).

Two major previous morphological investigations on the effects of a 4 h exposure to 3 ppm ozone on rat lungs are worth mentioning (81, 82). The epithelial damage was located centrally showing cell death of bronchiolar epithelium and alveolar pneumonocytes. Lesions in capillary endothelium with endothelial swelling were widespread with ring-like formations of endothelial membranes peripherally with alveolar and interstitial edema. These results show that ozone damage is in the centro-acinar regions and affects both endothelial and epithelial cells.

## Interleukins With Inflammatory or Protective Roles

**Figure 3:**
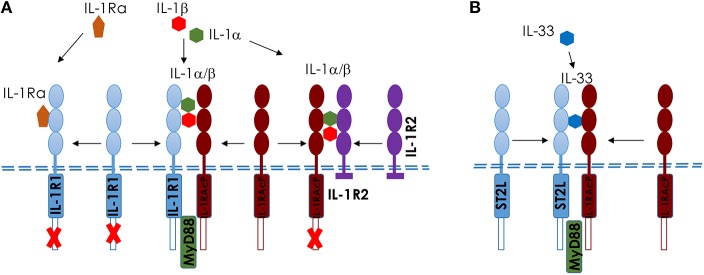
Comparison of IL-1 and IL-33 and their membrane receptors in mice. Scheme of IL-1 **(A)** and IL-33 **(B)**, showing the different ligands and receptors. **(A)** IL-1αβ binds to IL-1R1 and induces a conformational change in the extracellular domain of IL-1R1, enabling its interaction with IL-1RAcP, which is required for intracellular signaling, including MyD88-dependent activation. IL-1Ra competes with IL-1αβ for the binding to IL-1R1; the binding of IL-1Ra to IL-1R1 prevents IL-1αβ binding, the recruitment of IL-1RAcP and the activation of intracellular signaling pathways. IL-1R2 acts as a decoy receptor on the cell surface to IL-1RAcP. **(B)** Interleukin−33 (IL−33) binds to its transmembrane receptor suppression of tumorigenicity 2 (ST2) and induces a conformational change that allows ST2 to interact with IL−1 receptor accessory protein (IL−1RAcP). Activation of ST2 and IL−1RAcP leads to the Toll/IL−1 receptor (TIR) domains clustering and the recruitment of signaling adaptor myeloid differentiation primary response protein 88 (MYD88) (23).

Upon ozone exposure, several interleukins (IL) are increased and may regulate inflammation. Here we focus on two IL-1 family members, IL-1a and IL-33 (23, 83–85), which are known as alarmins, released upon cell stress and death. An overview of IL1 members and IL-33 and their receptors is shown in Figure 3.

## Interleukin-1 Enhancing Inflammation

A role of interleukin-1 (IL-1)-associated cytokines has been reviewed recently (24). We investigated inflammation after a single ozone exposure (1 ppm for 1 h) using IL-1α-, IL-1β-, and IL-18-deficient mice or neutralizing anti-IL-1α antibody to investigate their role in epithelial cell death. IL-1α was increased within 1 h after ozone exposure. Epithelial injury, inflammation and AHR were IL-1α-dependent. Further, we found that IL-1α signaling via IL-1R1/MyD88 was type I alveolar epithelial cell dependent as demonstrated by cell specific MyD88 deletion in mice (86). By contrast, inflammation and epithelial injury were less reduced in absence of IL-1β and IL-18. In conclusion, the IL-1α induced tissue damage and inflammation is mediated by IL-1R/MyD88 signaling in epithelial cells. Interestingly, in more chronic disease settings, IL-1β is increased in the lungs of mice with neutrophilic phenotype of asthma and in patients with neutrophilic asthma (27). Additionally, in humans IL-1β may lead to impairment of epithelial barrier function and increase of mucus production (27). Therefore, IL-1α or IL-1 receptor may represent a therapeutic target to attenuate ozone-induced lung inflammation and airway hyperreactivity. The use of neutralizing IL-1β antibody and IL1-RA antagonist is in fact used for different experimental conditions.

## Interleukin-33 Attenuating Inflammation

IL-33 is another alarmin released rapidly upon ozone and may have a protective role. IL-33 has homeostatic functions and is involved in injury and repair. The alarmin IL-33 is expressed at steady state in tissue cells and released upon airway epithelial injury and repair during inflammatory process (87, 88). IL-33 has functions in both innate and adaptive immune response (85). IL-33 binds to ST2 chain known as IL-33R or IL-1RL1 (89) which associates with IL-1RAcP (23). The full length, 35 kDa IL-33 is cleaved by different proteases and is produced by neutrophils, macrophages, or mast cells that full length IL-33 to several active moieties that are about 30-fold more active than the full length form (84, 90, 91). IL-33 receptor ST2 is was first detected in high endothelial venules in lymphoid tissues in mice (92), but is found in most innate immune cells including mast cells (85), innate lymphoid cells (85), myeloid and dendritic cells (93), and to a lesser extent on Th2 cells (85). Further, nuclear IL-33 may associate with chromatin *in vivo*, but the function of nuclear IL-33 is still unresolved.

IL-33 expression was increased in epithelial and macrophages and other myeloid cells in mice upon ozone exposure. In the absence of IL-33 or IL-33R/ST2, epithelial cell injury with protein leak and myeloid cell recruitment and inflammation was further increased, while tight junction proteins E-cadherin and ZO-1, ROS expression in neutrophils, and AHR were diminished (43). ST2 antibody neutralization recapitulated the enhanced ozone induced neutrophilic inflammation, while the administration of rmIL-33 reduced neutrophil recruitment in IL-33 deficient mice (69). These data demonstrate that ozone causes an immediate barrier injury, which precedes myeloid cell mediated inflammatory injury under the control of the IL-33/ST2 axis. Thus, IL-33/ST2 signaling appears to be critical for the maintenance of intact epithelial barrier and inflammation.

## Surfactant Proteins and Mucus

Surfactant proteins mucus are the first line protection protecting the respiratory epithelium. Surfactant protein-D (SP-D), produced by the airway epithelium, is multimeric protein sensitive to oxidative stress. SP-D directly inhibits extracellular DNA trap formation by eosinophils. Allergic airway sensitization and ozone exposure augmented eosinophilia and nos2 mRNA (iNOS) activation in the lung tissue with modification of SP-D in the airways. Thus, the regulatory feedback between SP-D and eosinophils appears to be destroyed by the NO-rich oxidative lung tissue environment in asthma exacerbations (94).

## Therapeutic Targets

The protective role of antioxidants against ROS/RNS induced injury and inflammation environmental pollutants has been reviewed (38, 95). A comprehensive analysis of oxidative stress in ozone induced lung injury and mitochondrial dysfunction allowed to identify potential druggable pathways (2, 38, 95, 96). Indeed, several interventions attenuate ozone induced inflammation such N-acetylcysteine (97), hydrogen disulfide (98), MIF antagonist (99) as well as blockade of IL-1α (86) or IL-17A (100), recombinant IL-33 (69), L-arginine promoting DNA repair (101), Taurine (102). Also, blocking ROS-induced airway inflammation by apocynin, an NADPH inhibitor has been targeted in asthma and COPD in human clinical trials (47, 103). Furthermore, DNA released upon cell death and is highly inflammatory. In particle-induced lung injury, enzymatic degradation of DNA by DNase I reduced inflammation (10, 11). The list therapeutic targets are not exhaustive, but new mechanistic insights may lead to antagonists that are more efficacious. However, reduction of airborne pollution, especially high levels of ozone and smog, would be the most efficacious measure to prevent chronic respiratory disease.

## Conclusions

Chronic ozone exposure causes chronic lung inflammation, emphysema, and interstitial fibrosis with progressive loss of lung function in man and rodents. Furthermore, ozone enhances the development of chronic lung diseases such as allergic and non-allergic asthma, COPD, and emphysema. Recent studies demonstrated the importance of IL-1α, IL-33, and IL-17A axis in ozone induced lung injury and inflammation, and a role of PGAM5 emerged, which defines a novel cell death pathway known as oxeiptosis. Understanding the fundamental mechanisms of injury and defective repair likely related to DNA damage, and defining critical targets require further investigations including clinical and epidemiological studies. Chronic progressive lung diseases could be prevented to a great extent by reducing environmental air pollution, tobacco, and wood fire smoke and smog containing ozone exposure.

## Author Contributions

MS, BR, and DT wrote the review. KC, VQ, and CA inspired the work and corrected. All authors read the contribution.

### Conflict of Interest Statement

DT is employee at ArtImmune. The remaining authors declare that the research was conducted in the absence of any commercial or financial relationships that could be construed as a potential conflict of interest.

## Abbreviations

AHR: airways hyperreactivity
AIFM1: mitochondrial apoptosis inducing factor 1
AJ: adherens junctions
AREG: amphiregulin
BAL: broncho alveolar lavage to quantify cell counts
BALF: broncho alveolar lavage fluid to detect protein levels
GR1: Ly-6G antibody depleting neutrophils
IL-33: interleukin-33
IL-33R/ST2: interleukin-33 receptor/ suppression of tumorigenicity 2
CXCL1/KC: keratinocyte chemoattractant
COPD: chronic obstructive pulmonary disease
LCN-2: lipocalin 2
CCL1/MCP-1: chemokine ligand 2
CCL2/MIP-2: chemokine (C-X-C motif) ligand 2
MMP-9: matrix metalloproteinase 9
MPO: myeloperoxidase
PGAM5: mitochondrial serine/threonine-protein phosphatase
rmIL-33: recombinant mouse IL-33
ROS: reactive oxygen species
RNS: reactive nitrogen species
TIMP-1: tissue inhibitor metalloproteinase 1
TJ: tight junctions
WT: wild type mice.
